# Supplementary material for: RNAmigos2: accelerated structure-based RNA virtual screening with deep graph learning
Source: Nat Commun. 2025 Mar 21;16:2799. doi: 10.1038/s41467-025-57852-0 (PMC11928640; doi:10.1038/s41467-025-57852-0)
Supplement: Supplementary file 2 — Reporting Summary [file 41467_2025_57852_MOESM2_ESM.pdf]

nature portfolio

Corresponding author(s): Carlos Oliver & Jérôme Waldispühl

Last updated by author(s): Jun 12, 2024

Reporting Summary

Nature Portfolio wishes to improve the reproducibility of the work that we publish. This form provides structure for consistency and transparency in reporting. For further information on Nature Portfolio policies, see our Editorial Policies and the Editorial Policy Checklist.

Statistics

For all statistical analyses, confirm that the following items are present in the figure legend, table legend, main text, or Methods section.

n/a	Confirmed	
☐	☒	The exact sample size (n) for each experimental group/condition, given as a discrete number and unit of measurement
☐	☒	A statement on whether measurements were taken from distinct samples or whether the same sample was measured repeatedly
☒	☐	The statistical test(s) used AND whether they are one- or two-sided<br>*Only common tests should be described solely by name; describe more complex techniques in the Methods section.*
☐	☒	A description of all covariates tested
☒	☐	A description of any assumptions or corrections, such as tests of normality and adjustment for multiple comparisons
☐	☒	A full description of the statistical parameters including central tendency (e.g. means) or other basic estimates (e.g. regression coefficient) AND variation (e.g. standard deviation) or associated estimates of uncertainty (e.g. confidence intervals)
☒	☐	For null hypothesis testing, the test statistic (e.g. F, t, r) with confidence intervals, effect sizes, degrees of freedom and P value noted<br>*Give P values as exact values whenever suitable.*
☒	☐	For Bayesian analysis, information on the choice of priors and Markov chain Monte Carlo settings
☒	☐	For hierarchical and complex designs, identification of the appropriate level for tests and full reporting of outcomes
☒	☐	Estimates of effect sizes (e.g. Cohen's d, Pearson's r), indicating how they were calculated

Our web collection on statistics for biologists contains articles on many of the points above.

Software and code

Policy information about availability of computer code

Data collection

All training data is taken directly from the RCSB-PDB Databank and annotated by x3dna-DSSR. Synthetic data was generated by running rDOCK on ligands collected from the RCSB-PDB as well as ChEMBL. Virtual screen validation was carried out using data from the ROBIN dataset which is publicly available at the following DOI: https://doi.org/10.1002/ange.202211358

Data analysis

All code used for analysis and was open source. Python packages used and versions when necessary are listed below:

torch==1.11.0
dgl==1.1.0
biopython==1.84
rdkit==Release_2024.09.1
rnaglib==2.0.4
git+https://github.com/cgoliver/fr3d-python.git

Code to use the proposed tool (rnamigos2) and reproduce all results is available on GitHub under an MIT license:

https://github.com/cgoliver/rnamigos2/

For manuscripts utilizing custom algorithms or software that are central to the research but not yet described in published literature, software must be made available to editors and reviewers. We strongly encourage code deposition in a community repository (e.g. GitHub). See the Nature Portfolio guidelines for submitting code & software for further information.

Data

Policy information about availability of data

All manuscripts must include a data availability statement. This statement should provide the following information, where applicable:

- Accession codes, unique identifiers, or web links for publicly available datasets
- A description of any restrictions on data availability
- For clinical datasets or third party data, please ensure that the statement adheres to our policy

All data used to train and test models is available as a sub-folder in our main repository at the following URL: https://github.com/cgoliver/rnamigos2/tree/master/data
All data generated in this study have been deposited in the Zenodo database under accession code 10.5281/zenodo.14803961. The data are available under the MIT license.

Research involving human participants, their data, or biological material

Policy information about studies with human participants or human data. See also policy information about sex, gender (identity/presentation), and sexual orientation and race, ethnicity and racism.

Reporting on sex and gender	n/a
Reporting on race, ethnicity, or other socially relevant groupings	n/a
Population characteristics	n/a
Recruitment	n/a
Ethics oversight	n/a

Note that full information on the approval of the study protocol must also be provided in the manuscript.

Field-specific reporting

Please select the one below that is the best fit for your research. If you are not sure, read the appropriate sections before making your selection.

☒ Life sciences ☐ Behavioural & social sciences ☐ Ecological, evolutionary & environmental sciences

For a reference copy of the document with all sections, see nature.com/documents/nr-reporting-summary-flat.pdf

Life sciences study design

All studies must disclose on these points even when the disclosure is negative.

Sample size	The main component of the work is a deep learning model. We collected the largest possible number of training points (RNA-small molecule binding sites) while enforcing a maximal structural similarity of 75% between the closest training and testing pockets. This resulted in 436 clusters of non-redundant binding sites.
Data exclusions	n/a
Replication	Models were re-trained 3 times using different random seeds.
Randomization	Binding sites were assigned randomly to training and testing splits greedily while ensuring the redundancy constraint is satisfied.
Blinding	n/a

Reporting for specific materials, systems and methods

We require information from authors about some types of materials, experimental systems and methods used in many studies. Here, indicate whether each material, system or method listed is relevant to your study. If you are not sure if a list item applies to your research, read the appropriate section before selecting a response.

Materials & experimental systems

n/a	Involved in the study
☒	☐ Antibodies
☒	☐ Eukaryotic cell lines
☒	☐ Palaeontology and archaeology
☒	☐ Animals and other organisms
☒	☐ Clinical data
☒	☐ Dual use research of concern
☒	☐ Plants

Methods

n/a	Involved in the study
☒	☐ ChIP-seq
☒	☐ Flow cytometry
☒	☐ MRI-based neuroimaging

Plants

Seed stocks	n/a
Novel plant genotypes	n/a
Authentication	n/a
